# Metabolic versatility of *Acinetobacter baumannii*: distinct pathways for the utilization of short-, medium- and long-chain acyl-L-carnitines

**DOI:** 10.3389/fmicb.2026.1964901

**Published:** 2026-09-18

**Authors:** Paula Schwerdhelm, Kateryna Podrez, Julian Kaltenhäuser, Simon Sivov, Beate Averhoff, Dominik Körner, Lothar Jänsch, Dieter Jahn, Jürgen Moser

**Affiliations:** 1 Institute of Microbiology, Technische Universität Braunschweig, Braunschweig, Germany; 2 Institute of Molecular Biosciences, Goethe-Universität Frankfurt, Frankfurt, Germany; 3 Department of Cellular Proteome Research, Helmholtz Centre for Infection Research, Braunschweig, Germany; 4 Braunschweig Centre of Integrated Systems Biology, Braunschweig, Germany

**Keywords:** *Acinetobacter baumannii*, acyl-L-carnitine, carnitine, hydrolase, trimethylamine

## Abstract

*Acinetobacter baumannii* is a multidrug-resistant pathogen capable of metabolizing host-derived acyl-L-carnitines, which contributes to its virulence. During *A. baumannii* growth on acetyl-L-carnitine, L-carnitine was initially released into the growth medium suggesting that acetate is metabolized first. The subsequent uptake of L-carnitine at the end of the exponential phase then facilitates the catabolism of L-carnitine. Involvement of the betaine/choline/carnitine transporter (BCCT) in the uptake of short- and medium-chain acyl-L-carnitines was confirmed through growth experiments using the Δ*bcct* mutant strain. An uncharacterized hydrolase (HMPREF0010_01348) encoded by the L-carnitine degradation gene operon (HMPREF0010_01345 – HMPREF0010_01351) was biochemically identified as a short- and medium-chain acyl-L-carnitine hydrolase (ACH). Its cellular localization in the cytoplasm was demonstrated. The purified ACH exhibited no enzymatic activity toward long-chain acyl-L-carnitines. However, growth experiments revealed that both the wild type and the Δ*ach* mutant were able to grow clearly on these substrates, providing evidence for the involvement of an additional, as-yet-uncharacterized enzyme. The cell culture supernatant was used for the chromatographic enrichment of a protein fraction showing robust enzyme activity for the hydrolysis of long-chain acyl-L-carnitines. Proteomic analysis of a partially purified hydrolase fraction and of the *A. baumannii* secretome revealed the gene product of open reading frame HMPREF0010_02333 as a hydrolase candidate. The respective protein was recombinantly overproduced and purified and its ability to cleave various long-chain acyl-L-carnitines was demonstrated. Based on the substrate specificity of the characterized ACH from the cytosol and the acyl-L-carnitine hydrolase present in the culture supernatant (ACHS), a metabolic model for the versatile utilization of small- medium- and long-chain acyl-L-carnitines by *A. baumannii* was proposed.

## 1 Introduction

*Acinetobacter baumannii* is a Gram-negative gammaproteobacterium that has been identified as a significant cause of nosocomial infections in healthcare institutions worldwide (Villegas and Hartstein, 2003). The opportunistic pathogen can cause pneumonia, meningitis and endocarditis as well as a wide range of skin, wound, soft tissue and urinary tract infections (Ibrahim et al., 2021). *A. baumannii* is known for developing high antibiotic resistance and the ability to survive under a wide range of environmental conditions. It is characterized by its outstanding resistance to desiccation and the ability to form biofilms (Harding et al., 2018). Furthermore, it possesses a high degree of metabolic flexibility, allowing it to utilize multiple host-derived carbon and nitrogen sources (Soares et al., 2009). The elucidation of the employed metabolic pathways might be important for developing future therapeutic strategies. A potentially crucial metabolic pathway in *A. baumannii* involves the catabolism of L-carnitine (Zhu et al., 2014; Breisch et al., 2022). This quaternary ammonium compound is abundant in host tissues, with concentrations of ~50 μM in blood, ~5 μmol/g in the heart and ~1.5 μmol/g in the liver (Hanai et al., 2020). L-carnitine primarily functions in humans as a shuttle for the translocation of long-chain fatty acids (as acyl-L-carnitines) into mitochondria for subsequent β-oxidation (Longo et al., 2016). *A. baumannii* can utilize L-carnitine as its exclusive source of carbon and energy (Zhu et al., 2014; Breisch et al., 2022).

Our laboratory has contributed to the detailed understanding of the L-carnitine-degrading gene cluster (Figure 1). It encodes for the LysR-type transcriptional activator called *carR*, the energy-dependent betaine/carnitine/choline transporter (*bcct*) and the two-component carnitine monooxygenase (*cntA*/*cntB*) which facilitates the intracellular cleavage of L-carnitine into trimethylamine and malic semialdehyde (Breisch et al., 2019; Massmig et al., 2020; Piskol et al., 2022). Malic semialdehyde dehydrogenase (*msadh*) leads to the formation of D-malate which is then converted into pyruvate by the encoded D-malate dehydrogenase (*mdh*) (Breisch et al., 2022; Piskol et al., 2024).

**Figure 1:**
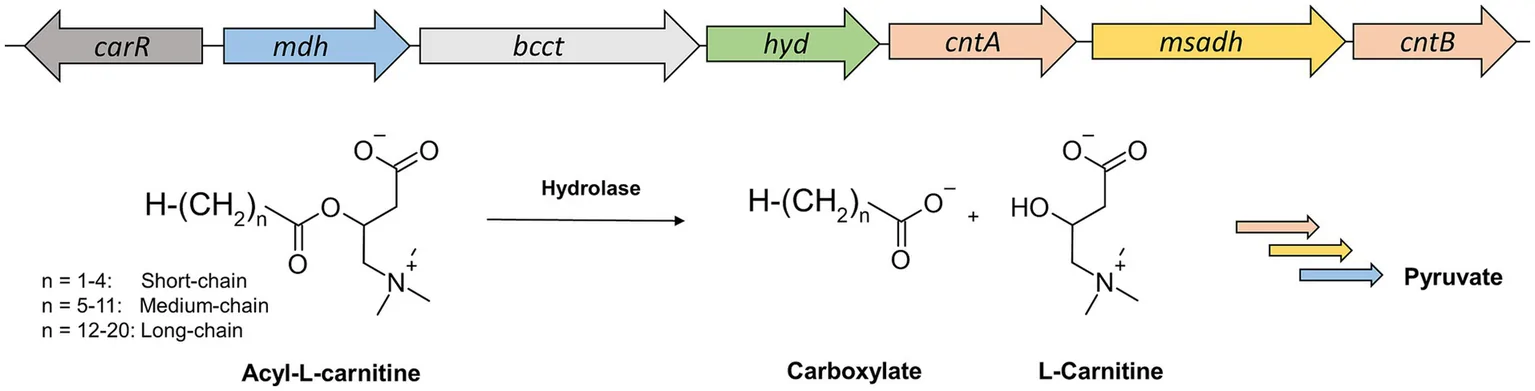
Proposed metabolism of acyl-L-carnitine in *A. baumannii*. Structure of the L-carnitine degradation gene operon (*top*) and the proposed metabolism of various acyl-L-carnitines (*bottom*). Genes *carR* (also termed *lysR*) and *bcct* encode for a LysR-type transcriptional activator and a transporter of the betaine/choline/carnitine family, respectively. Genes *cntA* and *cntB* encode for the subunits of carnitine monooxygenase, *msadh* for malic semialdehyde dehydrogenase, and *mdh* for D-malate dehydrogenase. Acyl-L-carnitines are mainly classified as short-chain, medium-chain and long-chain acyl-L-carnitines. Cleavage of these molecules yields the corresponding carboxylate and L-carnitine, which is further metabolized into pyruvate. The *hyd* gene likely encodes an acyl-L-carnitine hydrolase.

To date, the molecular function of the protein encoded by the *hyd* gene has not yet been elucidated. The respective deletion mutant strain (Δ*hyd*) exhibited impaired growth on acetyl-L-carnitine as the sole carbon and energy source (Breisch et al., 2022). Therefore, it was concluded that *hyd* encodes for a hydrolase that catalyzes the release of L-carnitine from acetyl-L-carnitine. The latter molecule belongs to the diverse group of acyl-L-carnitines which are mainly categorized as short-chain acyl-L-carnitines (C2-C5 acyl moiety), medium-chain acyl-L-carnitines (C6-C12 acyl moiety) and long-chain acyl-L-carnitines (C13-C21 acyl moiety). In mice, the highest acyl-L-carnitine content was observed in the heart (390–430 nmol/g), followed by muscles (250–350 nmol/g) and the lowest contents were observed in plasma (10–25 μM) and in adipose tissues (6–8 nmol/g) (Makrecka-Kuka et al., 2017). The metabolism of acyl-L-carnitine might play an important role for the virulence of *A. baumannii*. Bacterial infection studies using *Galleria mellonella* caterpillar in the presence of acetyl-L-carnitine revealed a significantly lower virulence for the Δ*hyd* mutant strain in comparison to the wild type (Breisch et al., 2022).

We therefore set out to elucidate the metabolic pathways underlying *A. baumannii* growth in the presence of different acyl-L-carnitine substrates. The hydrolase encoded by the *hyd* gene was recombinantly overproduced and purified, and its substrate range was characterized using a newly established acyl-L-carnitine hydrolase assay. Mutant strains were employed to investigate cellular substrate uptake and to examine the sequential utilization of the hydrolysis products. Finally, we report the identification and characterization of a second acyl-L-carnitine hydrolase showing complementary substrate specificity.

## 2 Materials and methods

### 2.1 Plasmid and strain construction

The *hyd* gene of *A. baumannii* ATCC 19606 (open reading frame HMPREF0010_01348) was synthesized as a codon optimized DNA fragment (GeneArt, Thermo Fisher) and was inserted into vector pETM11 (Dummler et al., 2005) via restriction sites *NcoI*/*NotI* yielding plasmid pETM11_*ach*. The construction of the markerless deletion mutants *A. baumannii* Δ*bcct* and Δ*hyd* was described elsewhere (Breisch et al., 2019; Breisch et al., 2022). The codon optimized gene for the postulated hydrolase (open reading frame HMPREF0010_02333) with an N-terminal pelB leader sequence from *Escherichia coli* was synthesized (GeneArt, Thermo Fisher) and inserted into the *NdeI*/*NotI* restriction sites of vector pETM50 (Dummler et al., 2005) yielding plasmid pETM50_pelB_*achs*. The gene for the carnitine dehydrogenase from *Pseudomonas aeruginosa* (cdhA, encoded by PA5386) was PCR amplified using primers 1 and 2 and inserted into plasmid pBAD_His_AM via *NcoI*/*NotI* cloning sites. The plasmid was named pBAD_His_AM_*cdhA*. Synthetic gene sequences and primer sequences are summarized in Supplementary Table S1.

### 2.2 Overproduction and purification of acyl-L-carnitine hydrolase

The target protein fused to an N-terminal His-tag was overproduced in *E. coli* Tuner (DE3) cells carrying plasmid pETM11_*ach*. An overnight culture was used to inoculate 6 × 500 mL of LB medium containing 50 μg mL^−1^ kanamycin. Cells were cultivated at 37 °C and 200 rpm in baffled flasks to an optical density at 578 nm of 0.5. Subsequently, protein production was initiated by addition of 50 μM isopropyl-β-d-thiogalactopyranoside (IPTG). After 20 h of cultivation at 17 °C and 180 rpm, the cells were harvested by centrifugation at 4,000 × g for 20 min at 4 °C. The resulting pellet was resuspended in 40 mL buffer 1 (100 mM HEPES-NaOH pH 7.5, 10 mM MgCl_2_, 150 mM NaCl) supplemented with 2.5 units/mL Turbo Nuclease (Jena Bioscience) and disrupted by a single passage through a french press at 16,000 psi. The lysate was clarified by centrifugation at 27,500 × g for 60 min at 4 °C and subsequent 0.45 μm filtration.

The soluble extract was applied to 2 mL Ni^2+^-loaded Ni-NTA Agarose (MACHEREY-NAGEL). The column was washed with 8 mL of buffer 1, and the target protein was subsequently eluted using 2 mL portions of buffer 1 containing 50, 100, 150 and 200 mM imidazole. Target protein fractions were identified by SDS-PAGE, dialyzed against buffer 1 and concentrated to ~6 mg mL^−1^ by ultrafiltration (Amicon Ultra, 30 kDa cut-off membrane, Merck Milipore).

### 2.3 Overproduction and purification of L-carnitine dehydrogenase

*E. coli* TOP10 cells harboring plasmid pBAD_His_AM_*cdhA* were employed for overproduction of N-terminally His-tagged L-carnitine dehydrogenase in 6 × 500 mL of LB medium supplemented with 100 μg mL^−1^ ampicillin. Cells were grown at 37 °C and 200 rpm in baffled flasks until reaching an optical density of 0.5 at 578 nm. Protein expression was then induced by addition of 0.002% (w/v) arabinose. Cells were harvested after 20 h of cultivation at 17 °C and 180 rpm. Affinity chromatography was carried out as described above in the presence of buffer 1 containing 10 mM imidazol.

### 2.4 Overproduction and purification of the hypothetical hydrolase

The theoretical hydrolase with an N-terminal pelB leader sequence and a C-terminal His-tag was overproduced in *E. coli* Tuner (DE3) cells (plasmid pETM50_pelB_*achs*). A preculture was used to inoculate 4 × 500 mL of LB medium containing 30 μg mL^−1^ kanamycin. Cells were grown in baffled flasks at 37 °C with shaking at 180 rpm until reaching an optical density of 0.5 at 600 nm. Production of the target protein was initiated by addition of 75 μM isopropyl-β-d-thiogalactopyranoside (IPTG). After 22 h of cultivation at 17 °C, the cells were harvested by centrifugation at 4,000 x g for 20 min at 4 °C. Cells were resuspended in 25 mL buffer 1 supplemented with 4 μL of Benzonase® (Jena Bioscience). Treatment with 2 mg/mL polymyxin B in the presence of 0.2 g mL^−1^ saccharose at 20 °C for 120 min induced the release of periplasmic contents. The periplasmic extract was obtained by centrifugation at 27,500 × g for 60 min at 4 °C, followed by filtration through a 0.45 μm membrane. This fraction was applied to a 5 mL HisTrap™ HP column (Cytiva) using an ÄKTA Explorer™ liquid chromatography system (Amersham Pharmacia Biotech). The column was washed with buffer 1 containing 20 mM imidazole. Proteins were liberated using a linear gradient from 20 to 500 mM imidazole in buffer 1. Target protein fractions were identified by SDS-PAGE and dialyzed against buffer 1. The concentration of purified proteins was determined using Bradford reagent (Sigma-Aldrich) according to the manufacturer’s instructions with bovine serum albumin as a standard.

### 2.5 UV–vis spectroscopy

Purified protein fractions at a concentration of 20–30 μM were subjected to UV–vis spectroscopy using a V-650 UV–Vis spectrophotometer (Jasco).

### 2.6 Analytical size exclusion chromatography

The native molecular mass of recombinant acyl-L-carnitine hydrolases was determined using analytical size exclusion chromatography. A Superdex 200 increase 5/150 GL column (GE Healthcare) was calibrated with protein standards (molecular weight marker kit MWGF200 and thyroglobulin and Apo-Ferritin from Sigma) in the presence of 50 mM HEPES-NaOH pH 7.5, 150 mM NaCl at a flow rate of 0.45 mL min^−1^. Samples of 50 μL at a concentration of 2 mg mL^−1^ were injected and the elution was monitored at 280 nm.

### 2.7 Measurement of acyl-L-carnitine hydrolase activity

The cleavage of different acyl-L-carnitines was measured using purified recombinant protein or (enriched) supernatant fractions of *A. baumannii* cell cultures. A microplate reader-based assay was utilized as recently outlined (Massmig et al., 2020), to quantify enzymatically liberated L-carnitine. The concentration of L-carnitine was measured using carnitine acetyltransferase and acetyl-CoA. The generated CoA enabled for the cleavage of 5,5′-dithiobis-(2-nitrobenzoic acid) (DTNB) and the resulting 2-thio-5-nitrobenzoic acid (TNB) was monitored spectroscopically.

A standard hydrolase assay in a volume of 150 μL was performed in the presence of 300 μM acyl-L-carnitine in buffer 2 (20 mM HEPES-NaOH pH 7.5, 150 mM NaCl, 10 mM MgCl_2_). Acyl-L-carnitines (Sigma) were previously dissolved in 20 mM HEPES pH 7.5 at a concentration of 3 mM and stored at −20 °C until use. The enzymatic reaction was initiated at 27 °C by addition of 200 nM recombinant enzyme or by supplementing 50 μL of a cell supernatant fraction. In all cases, a negative control with heat-inactivated hydrolase (or supernatant fraction) was processed accordingly. At indicated time points, 25 μL of the assay mixture were removed and the liberated L-carnitine content was quantified by measuring the absorption at 412 nm. Defined L-carnitine concentrations in H_2_O were used to determine a calibration curve (0–200 μM). Absorbance values exhibited a slight dependence on the sample protein content, as Ellman’s reagent reacts with all free SH groups. However, this did not affect the calculation of linear slopes used to determine enzyme activity.

A coupled lauroyl-L-carnitine hydrolase assay was newly established for the kinetic investigation of potential acyl-L-carnitine hydrolases from the cell supernatant. Therefore, a continuous spectroscopic assay in the presence of purified L-carnitine dehydrogenase (CdhA, encoded by PA5386 from *Pseudomonas aeruginosa*) was set up to determine the initial rate of hydrolase activity, by monitoring the formation of NADH at 340 nm. A typical assay in a volume of 300 μL was performed in the presence of 300 μM acyl-L-carnitine, 0.7% (v/v) Triton X100 in buffer 1 containing 25–160 nM of the purified hypothetical hydrolase, 2 mM NAD^+^ and 1.6 μM of the recombinantly purified L-carnitine dehydrogenase at 27 °C. The enzymatic liberation of L-carnitine from different acyl-L-carnitines was quantified using a calibration curve that relates absorption to L-carnitine concentrations. Negative controls in the absence of hydrolase were analyzed accordingly. The measurements for the negative control exhibited a certain degree of variability due to the Triton X 100 used. Therefore, the mean of all absorption measurements was subtracted as the blank. All kinetic measurements were performed in triplicate and repeated at least two times.

### 2.8 Localization studies

The subcellular localization of the acyl-L-carnitine hydrolase activity in *A. baumannii* was determined by investigating potential enzyme activity in the culture supernatant (see below), the periplasmic fraction or the cytosolic fraction.

The separation of the cytosolic and periplasmic fractions was achieved through the formation of sphaeroblasts (Scribano et al., 2019) according to the following procedure. A 500 mL cultures of the wild type (or of the Δ*hyd* mutant strain) was cultivated on acetate (5 mM) until the late exponential phase. Cells were harvested and washed in 30 mM Tris–HCl pH 8.0. The pellet was suspended in 1 mL of 30 mM Tris–HCl buffer containing 20% w/v sucrose. Subsequently, 100 μL of lysozyme (100 mg mL^−1^) and 100 μL of EDTA (0.1 M) were added at room temperature, and sphaeroblast formation was examined using a phase-contrast microscope. After ~15 min, the sphaeroblasts were sedimented at 3,000 x g for 15 min and the resulting periplasmic fraction was collected. Sphaeroblasts were disrupted using a Fast Prep-24 (MP Biomedicals). The supernatant after centrifugation (13,000 rpm at 4 °C for 10 min) contained the cytosolic extract. The periplasmic or the cytosolic fraction were filtered sterile and dialyzed against buffer 1 for subsequent enzyme activity assays.

### 2.9 Growth curves and L-carnitine determination in the culture supernatant

The growth characteristics of wild type *A. baumannii* and of the Δ*bcct* or Δ*hyd* mutant strain was assessed on MOPS minimal medium (Neidhardt et al., 1974) which was supplemented with different acyl-L-carnitines (5 mM) or acetate (5 mM) as carbon and energy source. Carry-over of media components into the main culture was prevented as follows. A pre-culture (10 mL) was cultivated on LB medium at 37 °C for ~20 h. Cells were harvested by centrifugation at 4,000 × g for 15 min at room temperature and the supernatant was discarded. The cell pellet was suspended in MOPS minimal medium containing, e.g., acetate, L-carnitine or the respective acyl-L-carnitines (5 mM). The resulting culture was grown for 16 h at 200 rpm and subsequently harvested. The cell pellet was suspended in 1 mL MOPS minimal medium and the optical density at 600 nm (OD_600_) was determined. This inoculum was utilized to prepare a master mix, which was then employed to initiate a series of growth experiments with a start OD_600_ of 0.15. Bacterial growth was monitored from 250 μL cultures in 96-well microplates and the OD_600_ was followed over 24 h using an Infinite 200 pro M Nano plate reader (Tecan) at 198 rpm and 37 °C (time intervals 10 min).

Furthermore, a series of 12 parallel cultivations were conducted to assess the extracellular L-carnitine concentration at regular time intervals of 1 h (and after 12 and 24 h). A 100 μL sample of the cell suspension was taken at each time point and subjected to centrifugation (4,000 × g for 10 min). The resulting upper layer was sterile filtered, and the L-carnitine content was subsequently determined in accordance with the above mentioned methodology. Growth curves and L-carnitine quantification from supernatants were conducted in triplicate and repeated at least two times. The wild type, the Δ*bcct* and the Δ*hyd* strain was processed accordingly.

### 2.10 Enrichment of acyl-L-carnitine hydrolase activity from the cell supernatant

Cultures of the wild type or of the Δ*hyd* mutant strain (2 × 500 mL, each) were grown to the late exponential phase (OD_600_ ~ 0.3) on minimal medium containing 5 mM acetate. Cells were sedimented at 4,000 × g for 20 min and the clarified culture supernatants were sterile-filtered (0.2 μm pore size). The protein-containing filtrates were concentrated to a volume of ~2 mL using Vivaspin 20 ultrafiltration devices (Sartorius) with a 10 kDa cut-off limit. Concentrated fractions were dialyzed against 20 mM HEPES-NaOH pH 7.5 (buffer 3) and acyl-L-carnitine hydrolase activity was determined. Anion exchange chromatography on a MonoQ 5/50 column was then performed using an Äkta Explorer liquid chromatography system (GE Healthcare). The column was equilibrated with three column volumes (CV) of buffer 3 at a flow rate of 0.5 mL min^−1^. Injection of ~1.8 mL of each sample was followed by a linear gradient of 0–1 M NaCl for 10 CV. Fractions of 0.5 mL were collected and acyl-L-carnitine hydrolase activity was determined.

### 2.11 Mass spectrometric identification of proteins from the *A. baumannii* secretome

Proteins were solubilized in 50 mM triethylammonium bicarbonate, then reduced with 5 mM tris(2-carboxyethyl)phosphine for 1 h at 56 °C and alkylated with 10 mM methyl methanethiosulfonate for 30 min at room temperature. Enzymatic digestion was performed overnight at 37 °C under continuous agitation at 800 rpm, using trypsin (Promega) at a ratio of 0.2 μg enzyme per 1 μg protein. The resulting peptides were vacuum-dried, resuspended in 50 μL 0.1% formic acid in water, and desalted using C18 ZipTips (Merck). After a second drying step, peptides were resuspended in 50 μL 0.1% formic acid in water prior to liquid chromatography–tandem mass spectrometry (LC–MS/MS) analysis.

Chromatographic separation and mass spectrometric detection was performed on a Dionex Ultimate 3,000 n-RSLC system (Thermo Fisher) coupled to an Orbitrap Fusion Tribrid (Thermo Fisher). A total amount of 200 ng peptide per sample was loaded onto a C18 trap column (Acclaim, 3 μm RP18, 0.075 × 20 mm), washed at 6 μL min^−1^ for 3 min. Peptide separation was achieved using a 2 μm C18 analytical column (Pharmafluidics) at a flow rate of 300 nL min^−1^ via a 30 min linear gradient from 96.3% MS buffer A (0.1% formic acid, 5% DMSO in water) to 31.3% MS buffer B (0.1% formic acid, 5% DMSO, 80% acetonitrile), followed by a 10 min gradient from 31.3 to 62.5% MS buffer B and a final 2 min gradient reaching 90% MS buffer B. The system was operated using Thermo Scientific SII software within the Xcalibur suite (v4.3.73.11, Thermo Fisher), and electrospray ionization was achieved via a stainless-steel emitter. Data-dependent acquisition was carried out in “top speed” mode with a 3 s time window, in which doubly and triply charged peptides were selected for subsequent fragmentation. Peptide fragmentation was achieved by higher-energy collisional dissociation, and peptides were detected in the ion trap.

Raw MS/MS files were searched against the *Acinetobacter baumannii* database (UniProt: UP000498640, 2024-12-23) using PEAKS Studio 11.0 with the following parameters: enzyme, trypsin; maximum missed cleavages, 2; fixed modification, β-methylthiolation (C, Δm = +45.99 Da); variable modification, oxidation (M, Δm = +15.99 Da); precursor and fragment mass tolerances of 15 ppm and 0.5 Da, respectively. The false discovery rate was set to 1%.

## 3 Results

### 3.1 The *hyd* gene of *A. baumannii* promotes growth on acetyl-L-carnitine

Recently, we elucidated the central function of the carnitine degradation cluster of *A. baumannii* (Piskol et al., 2024), but the detailed role of the *hyd* gene for the metabolism on different acyl-L-carnitine compounds remains to be resolved. This gene is preceded by the coding sequence of the carnitine transporter *bcct* and is followed by the carnitine monooxygenase gene *cntA*. Deletion of the *hyd* gene yielded an *A. baumannii* mutant strain (Δ*hyd*) which exhibited impaired growth when acetyl-L-carnitine was the sole carbon and energy source. It was concluded that *hyd* encodes for a hydrolase that liberates L-carnitine from acetyl-L-carnitine. The Δ*bcct* mutant was likewise unable to grow on acetyl-L-carnitine, suggesting that acetyl-L-carnitine utilization of *A. baumannii* requires the *hyd* gene product in conjunction with the BCCT transporter (Breisch et al., 2022). These experimental findings were independently reproduced using microplate reader-based growth assays (Supplementary Figure S1).

### 3.2 Recombinant production and purification of the acyl-L-carnitine hydrolase from *A. baumannii*

To identify the substrate spectrum of the proposed hydrolase, we recombinantly overproduced the respective enzyme as an N-terminal His-tagged protein in *E. coli*. Ni^2+^-loaded chelating sepharose was used for the chromatographic purification which yielded almost homogeneous protein fractions with a relative molecular mass of ∼40,000 Da as judged by SDS-PAGE (calculated molecular mass, 39,532 Da) (Figure 2A). Approximately 27 mg of the purified target protein was obtained per liter cell culture.

**Figure 2:**
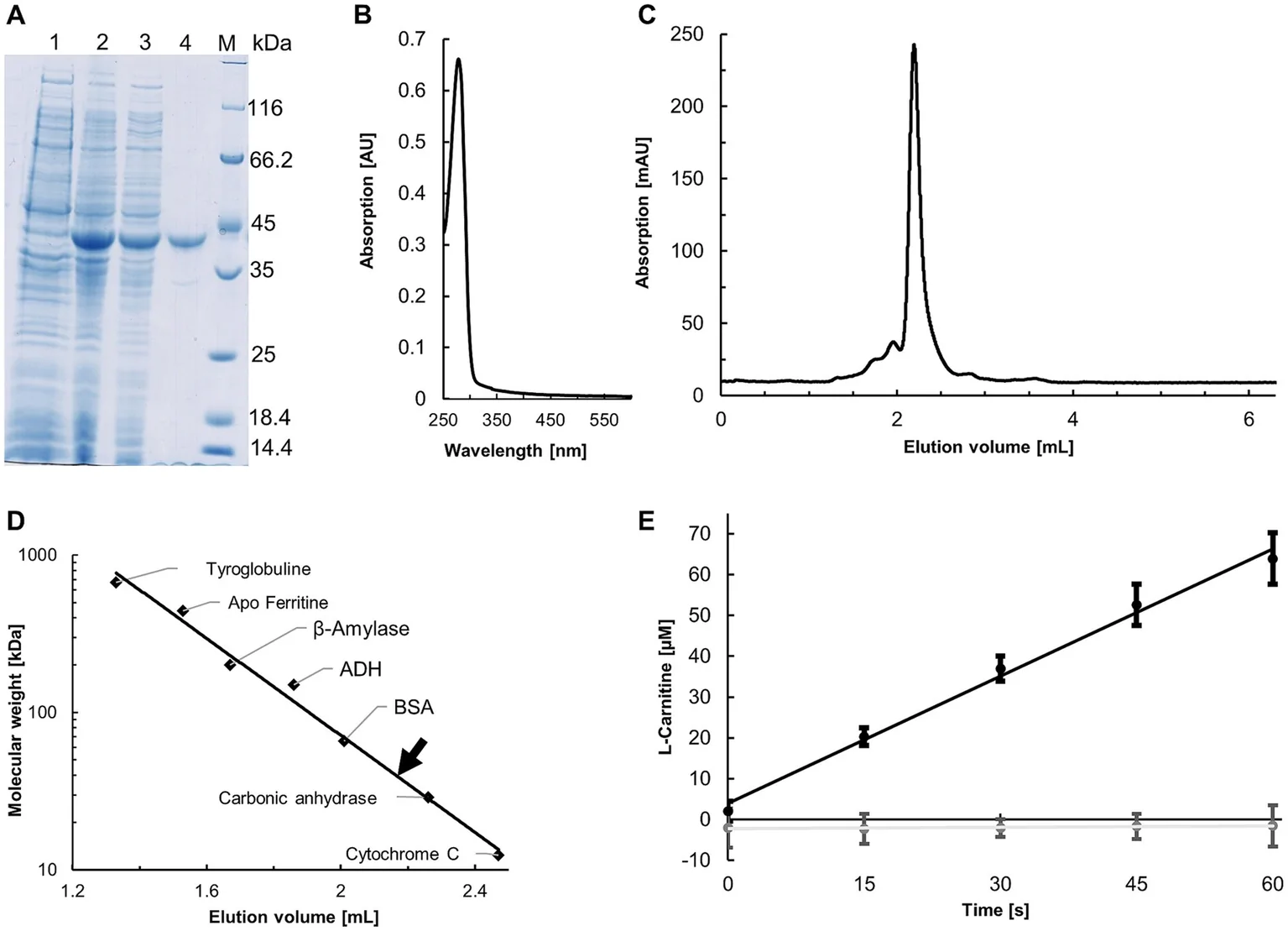
Purification and characterization of acyl-L-carnitine hydrolase (ACH). **(A)** SDS-PAGE analysis of the affinity purification of acyl-L-carnitine hydrolase. *Lanes 1 and 2*, *E. coli* cells before *and* after IPTG induction; *lane 3*, cell-free extract; *lane 4*, elution fraction after cell disruption, ultracentrifugation and affinity chromatography; *lane M*, molecular mass marker, relative molecular masses are indicated. **(B)** Absorption spectrum of the purified protein. **(C,D)** Native molecular mass determination of ACH by analytical gel filtration. Purified ACH was analyzed on a Superdex 200 increase 5/150 GL column at a flow rate of 0.45 mL min^−1^ monitoring the absorption at 280 nm. The column was calibrated with thyroglobulin (Mr = 660,000), apoferritin (Mr = 440,000), β-amylase (Mr = 200,000), alcohol dehydrogenase (ADH, Mr. = 150,000), albumin (BSA, Mr. = 66,000), carbonic anhydrase (Mr = 29,000) and cytochrome c (Mr = 12,400). The molecular mass of ACH is indicated by a black arrowhead. **(E)** Acyl-L-carnitine hydrolase assay containing purified ACH and acetyl-L-carnitine. The liberated L-carnitine was quantified colorimetrically in a coupled carnitine acetyltransferase reaction (*black line*). The specific activity was determined from the slope of the resulting curve. A control reaction with heat inactivated ACH was performed accordingly (*gray line*). The detection method used quantifies thiol groups non-specifically, so the initial L-carnitine concentration depends slightly on the protein composition of the sample. Each point represents the mean of three independent experiments. Error bars represent the standard deviation.

UV–visible absorption spectroscopy of concentrated protein samples did not show the presence of a chromophoric cofactor (Figure 2B). Analytical size exclusion chromatography revealed a native molecular mass of 38.5 kDa (Figures 2C,D), indicative for a monomeric quaternary structure.

### 3.3 Acyl-L-carnitine hydrolase assay

A microplate reader-based hydrolase assay was established which allowed to quantify the liberated L-carnitine from a range of acyl-L-carnitine compounds. L-carnitine concentrations were determined in the presence of carnitine acetyltransferase and acetyl-CoA as recently detailed (Massmig et al., 2020). The emergence of CoA resulted in the cleavage of 5,5′-dithiobis-(2-nitrobenzoic acid) (DTNB), and the resulting 2-thio-5-nitrobenzoic acid (TNB) was monitored spectroscopically. The activity assay containing the substrate acetyl-L-carnitine (300 μM) in the presence of the recombinant hydrolase (200 nM) revealed a specific hydrolase activity of 8.70 ± 0.58 μmol mg^−1^ min^−1^ (Figure 2E, *black line*). The heat-inactivated enzyme was used in control experiments (*gray line*).

### 3.4 *In vitro* conversion of acyl-L-carnitine substrates with varying acyl chain lengths

Besides acetyl-L-carnitine, the related compounds butyryl-L-carnitine (C4; small-chain), hexanoyl-L-carnitine, octanoyl-L-carnitine, lauroyl-L-carnitine (C6, C8, C12; medium-chain) and palmitoyl-L-carnitine (C16, long-chain) were also considered as hydrolase substrates (Supplementary Figure S2). Butyryl-L-carnitine, octanoy-L-carnitine and lauroyl-L-carnitine revealed substantially increased specific activities of 15.41 ± 2.58, 22.73 ± 0.91 and 11.54 ± 1.12 μmol mg^−1^ min^−1^ when compared to acetyl-L-carnitine. The highest activity of 42.64 ± 2.86 μmol mg^−1^ min^−1^ was determined in the presence of hexanoyl-L-carnitine. By contrast, the employed enzyme assay did not indicate any detectable activity in the presence of palmitoyl-L-carnitine, even in the presence of elevated enzyme concentrations of 500 nM. The *green* bars in Figure 3 illustrate the enzyme activity profile with respect to the individual substrate. Based on these *in vitro* data, the enzyme was designated as a short and medium-chain acyl-L-carnitine hydrolase which is hereinafter referred to as ACH.

**Figure 3:**
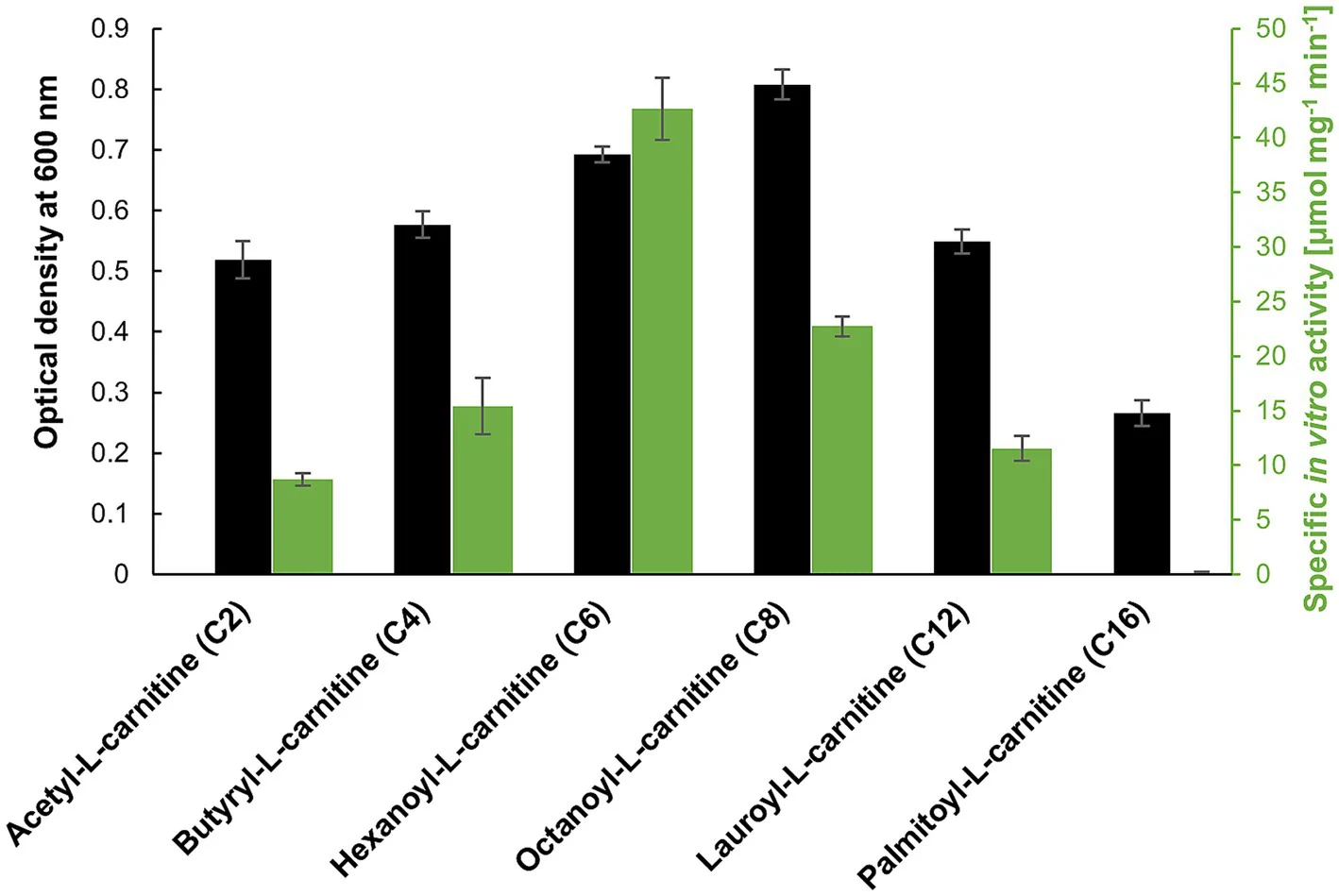
*In vivo* and *in vitro* conversion of acyl-L-carnitines with varying acyl chain length. Growth of *A. baumannii* on minimal medium containing different acyl-L-carnitine substrates was analyzed by comparing the achieved optical densities (OD_600_ values) after 16 h (*black bars*). The specific activity of purified ACH in the presence of the indicated substrates was calculated (*green bars*). Each data point corresponds to the mean of three independent experiments, with error bars representing the standard deviation. Purified ACH did not reveal detectable enzymatic activity in the presence of palmitoyl-L-carnitine. Concurrently, bacterial growth was clearly observed on this substrate.

These *in vitro* findings suggest that acyl-L-carnitine compounds with different acyl-chain lengths might contribute to the metabolism of *A. baumannii*. However, these results do not provide sufficient evidence for the understanding of the acyl-L-carnitine metabolism on the cellular level. Accordingly, bacterial growth experiments on minimal medium supplemented with the respective acyl-L-carnitines were conducted.

### 3.5 *A. baumannii* grows on various acyl-L-carnitines

Growth of *A. baumannii* on the different acyl-L-carnitine substrates was assessed by comparing the achieved optical densities (OD_600_ values) after 16 h (Figure 3, *black* bars and Supplementary Figure S1). In agreement with the preceding *in vitro* experiments, the wild type strain revealed substantial growth on acetyl-L-carnitine (0.52 ± 0.03) butyryl-L-carnitine (0.57 ± 0.02), hexanoyl-L-carnitine (0.69 ± 0.01), octanoyl-L-carnitine (0.81 ± 0.03) and lauroyl-L-carnitine (0.55 ± 0.02). To a certain extent, the bacterial growth experiments reflect the results of the enzyme kinetics, with maximum values being obtained for substrates hexanoyl-L-carnitine and octanoyl-L-carnitine, respectively. However, a less pronounced decline in cellular growth was evident with the other substrates.

The outcome of the growth experiments conducted in the presence of palmitoyl-L-carnitine were found to be in stark opposition to the results of the related enzyme kinetics. This long-chain acyl-L-carnitine clearly enabled for *A. baumannii* growth (0.26 ± 0.02) whereas no enzymatic activity was detectable in the corresponding *in vitro* experiment. This was taken as an initial indication that there may be a difference in the metabolic processing of larger substrates.

### 3.6 Localization of acyl-L-carnitine hydrolase in the cytosol

The subcellular localization of the *A. baumannii* ACH enzyme is of central importance for the understanding of the overall acyl-L-carnitine metabolism. Bioinformatics analyses using SignalP 6.0 (Teufel et al., 2022) suggested a cytosolic localization, as no signal sequence for the translocation of ACH was identified. To confirm ACH as a cytosolic protein, subcellular fractionation experiments from wild type *A. baumannii* cells in combination with enzyme activity studies were performed. Potential ACH activity (using acetyl-L-carnitine as a substrate) was assessed from the following samples. The culture supernatant from a 1 L cultivation was sterile filtered und subsequently concentrated to a volume of ~2 mL by ultrafiltration. No detectable ACH enzyme activity was determined for the extracellular fraction. Alternatively, a periplasmic fraction was prepared by lysozyme and EDTA treatment which solely resulted in a minor acetyl-L-carnitine hydrolysis activity of 0.98 ± 1.63 nmol min^−1^ mL^−1^. Following the mechanical disruption of the remaining sphaeroblasts, the resulting cytosolic extract exhibited a high ACH activity of 77.04 ± 11.63 nmol min^−1^ mL^−1^. These findings clearly indicate that ACH of *A. baumannii* is a soluble cytosolic protein.

### 3.7 Uptake of specific acyl-L-carnitines via the BCCT transporter

The ACH-dependent conversion of acyl-L-carnitines requires the initial translocation of the respective substrates into the cytosol. The ACH-depleted *A. baumannii* strain Δ*hyd* in combination with the transporter mutant strain Δ*bcct* was used to delineate the genetic prerequisites for the conversion of individual substrates (Figure 4 and Supplementary Figure S1).

**Figure 4:**
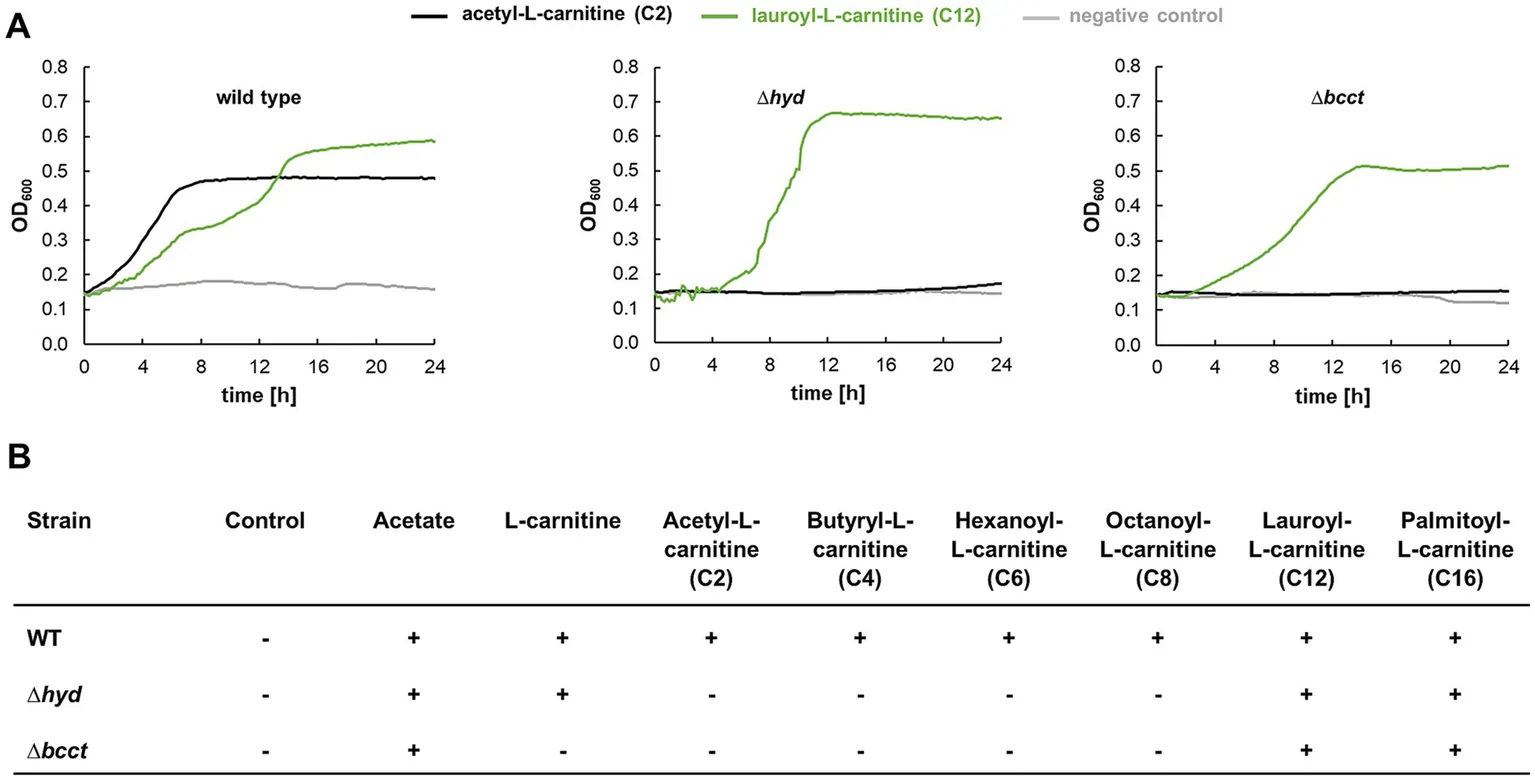
Bacterial growth of mutant strains Δ*hyd* and Δ*bcct* on acetyl-L-carnitines with varying acyl chain length. **(A)** Growth curves of wild type and mutant strains Δ*hyd* and Δ*bcct* on minimal medium containing acetyl-L-carnitine (*black curves*) and lauroyl-L-carnitine (*green curves*). Control experiments without bacterial inoculation were performed in parallel (*gray curves*). **(B)** Identical experiments as in **(A)** were also performed in the presence of acetate, L-carnitine, butyryl-L-carnitine, hexanoyl-L-carnitine, octanoyl-L-carnitine, and palmitoyl-L-carnitine. The table summarizes the results of the overall mutant analysis, with (−) indicating no bacterial growth and (+) indicating growth (OD_600_ increase > 0.1). All experiments were performed in triplicate and independently reproduced at least twice.

As anticipated, strain Δ*hyd* demonstrated growth in the presence of L-carnitine, but not in the presence of acetyl-L-carnitine, butyryl-L-carnitine, hexanoyl-L-carnitine and octanoyl-L-carnitine. The Δ*bcct* mutant was unable to grow on L-carnitine which might indicate that this substrate is transported via the BCCT Transporter, as previously described (Breisch et al., 2019). At the same time, no growth of this strain was observed on acetyl-L-carnitine, butyryl-L-carnitine, hexanoyl-L-carnitine and octanoyl-L-carnitine. This suggests, that the transport of these acyl-L-carnitines into the cytosol may also be facilitated by the BCCT transporter.

### 3.8 A distinct pathway for larger acyl-L-carnitines?

The growth experiments conducted on strains Δ*hyd* and Δ*bcct* in the presence of lauroyl-L-carnitine and palmitoyl-L-carnitine yielded unexpected yet consistent results. Both deletion strains exhibited substantial bacterial growth on both substrates (Figure 4). These findings lead to two conclusions: (I) The metabolism of larger acyl-L-carnitines is not mediated by the intracellular ACH enzyme, and (II) Both substrates can be metabolized in the absence of the BCCT transporter. The presence of an additional non-cytoplasmic acyl-L-carnitine hydrolase with a pronounced specificity for larger substrates would explain these findings. This hypothesis aligns with our initial ACH *in vitro* enzyme activity measurements, particularly in the presence of palmitoyl-L-carnitine. Notably, the recombinant ACH enzyme did not utilize this larger molecule as a substrate, supporting the notion of an additional hydrolase with distinctly different substrate specificity.

### 3.9 Cytosolic hydrolysis of acetyl-L-carnitine is linked to L-carnitine secretion

Acetyl-L-carnitine cleavage also results in the formation of acetate which can be converted into acetyl-CoA in *A. baumannii* (Huang et al., 2024). According to this, bacterial growth of wild type (and mutant strains) on acetate as a sole carbon and energy source was determined (Figure 4B and Supplementary Figure S2).

To further elucidate the versatile metabolism of *A. baumannii*, bacterial cultures were cultivated in the presence of acetyl-L-carnitine and the extracellular L-carnitine content was determined from culture supernatants. Figure 5A shows the optical density (OD_600_) of the wild type strain and the extracellular L-carnitine concentration plotted over time. The initial lag phase does not result in the release of substantial amounts of L-carnitine. However, the first half of the exponential growth is accompanied by a rapid increase of the extracellular L-carnitine concentration which reaches a maximum of ~ 330 μM at ~ 6 h. This increasing phase is followed by steep decline phase which corresponds to the second half of the exponential phase. No residual L-carnitine was detectable at ~10 h when the cell culture entered the stationary phase.

**Figure 5:**
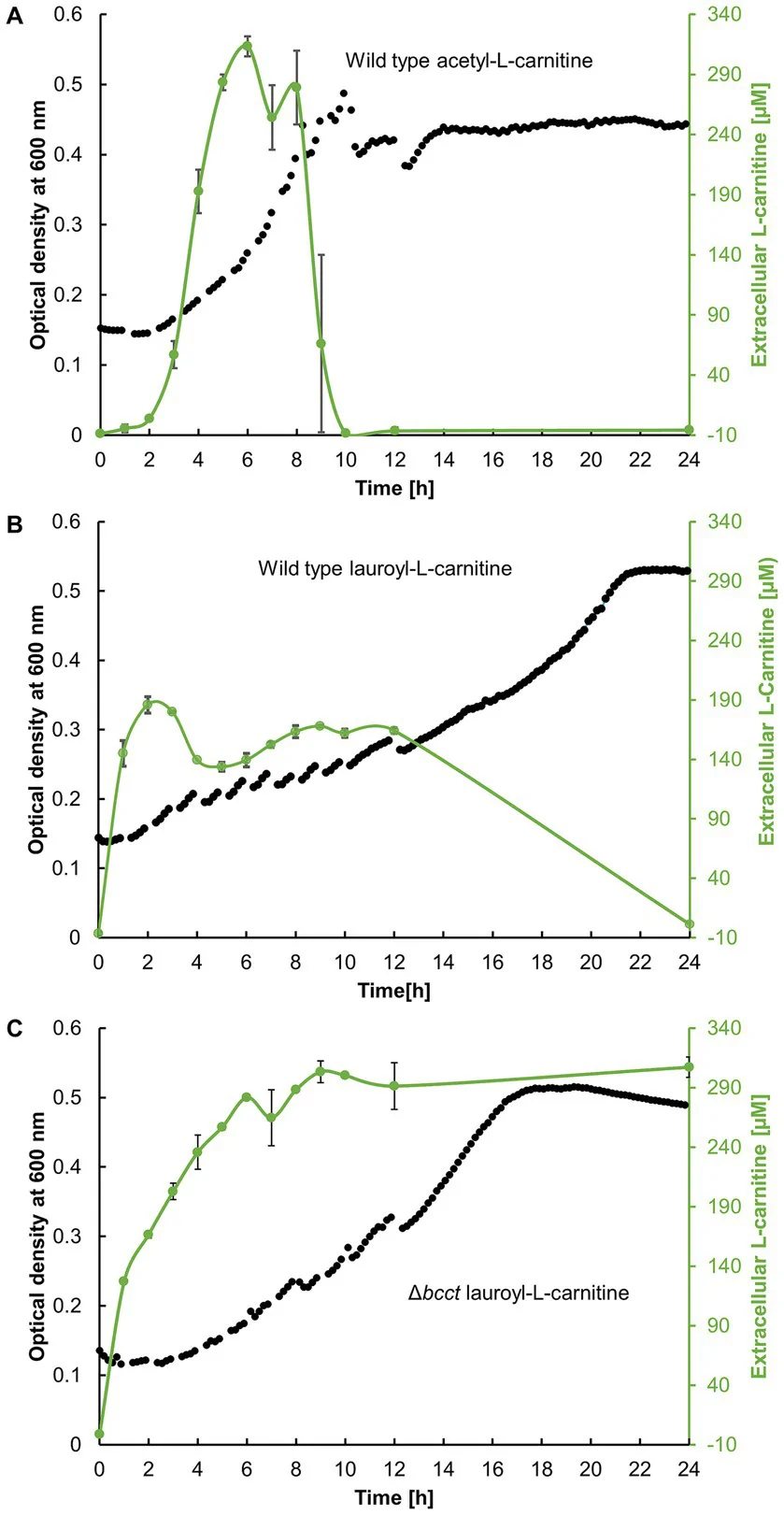
L-carnitine levels in supernatants of wild type and Δ*bcct* cultures grown on acetyl-L-carnitine and lauroyl-L-carnitine. **(A)** Time course of extracellular L-carnitine concentrations in a wild type culture grown in minimal medium containing acetyl-L-carnitine. **(B)** Time course of extracellular L-carnitine concentrations in a wild type culture grown in minimal medium containing lauroyl-L-carnitine. **(C)** Time course of extracellular L-carnitine concentrations in a Δ*bcct* culture grown in minimal medium containing lauroyl-L-carnitine. **(A–C)** The L-carnitine concentration in the culture supernatant was quantified colorimetrically (*green curve*). Each point represents the mean of three independent experiments and error bars represent the standard deviation. The progression of the optical density (OD_600_ values) is indicated (*black dots*). Growth curves were conducted in triplicate and reproduced at least twice.

Due to the intracellular cleavage of acetyl-L-carnitine it becomes evident that *A. baumannii* facilitates the secretion of L-carnitine. This might be indicative of a metabolic preference for acetate. Given its role as a compatible solute, the intracellularly generated L-carnitine must be efficiently secreted into the minimal medium during the initial growth phase. In the second half of the exponential phase, uptake of L-carnitine from the cell supernatant via the BCCT transporter takes place which then enables the intracellular metabolism of this compound as a carbon and energy source.

### 3.10 Lauroyl-L-carnitine cleavage in the lag phase

Lauroyl-L-carnitine was chosen as a model substrate for the investigation of the metabolism of larger acyl-L-carnitines due to its favorable solubility properties. Figure 5B shows the growth curve of the wild type strain with the time course of the extracellular L-carnitine content. After just 2 h, the L-carnitine concentration of the medium reached a maximum value of ~180 μM, although no significant cellular growth took place during this phase.

The same type of analysis was also performed with the transporter deficient mutant Δ*bcct* (Figure 5C). The growth experiment in the presence of lauroyl-L-carnitine resulted in a substantially higher L-carnitine concentration in the cell supernatant of ~ 300 μM. This high concentration remained at this high level throughout the 24-h experimental period. These findings might indicate that the metabolism of L-carnitine is abolished in the absence of the BCCT transporter. Laurate might be synthesized from lauroyl-L-carnitine and subsequently metabolized by the β-oxidation pathway of *A. baumannii* (Jiang et al., 2019). In line with these findings, extracellular cleavage of lauroyl-L-carnitine, e.g., by a secreted hydrolase was hypothesized.

### 3.11 Lauroyl-L-carnitine hydrolase activity in the supernatant of *A. baumannii*

A wild type culture of 1 L was grown on acetate and the cell supernatant after centrifugation was sterile filtered and subsequently concentrated by ultrafiltration employing a membrane with a 10 kDa cut-off limit. A sample of this retentate was subjected to a lauroyl-L-carnitine hydrolase assay using a substrate concentration of 300 μM. A specific cleavage activity of 9.9 ± 5.1 nmol min^−1^ mL^−1^ was determined. A negative control with a heat inactivated sample did not reveal detectable activity. The same experiment was also conducted from the supernatant of the ACH deleted mutant strain Δ*hyd.* A clear activity of 30.1 ± 0.1 nmol min^−1^ mL^−1^ in the presence of lauroyl-L-carnitine was observed. Concentrated supernatant fractions (from wild type and Δ*hyd*) did not indicate detectable activity when the small-chain molecule acetyl-L-carnitine was used as a substrate.

These findings clearly indicate that an additional hydrolase activity is present in the soluble supernatant of *A. baumannii*. The as yet undescribed ACH in the cell supernatant was termed ACHS. This enzyme possesses a high specificity for larger acyl-L-carnitine substrates.

The initial experiments were conducted on a large-scale culture that had been cultivated on acetate. Thus, the observed enzyme activity was expressed constitutively in the absence of the lauroyl-L-carnitine substrate. A hydrolase activity assay was also conducted on the basis of a small-scale culture of wild type cells which had been cultivated on lauroyl-L-carnitine. The respective culture supernatant, without a sample concentration step, clearly revealed an enzymatic activity of 1.7 ± 0.4 μmol min^−1^ mL^−1^. The same type of experiment with an acetate grown culture did not indicate enzyme activity above the detection limit of the employed enzyme assay. The results clearly show that the hydrolase to be identified is induced in the presence of lauroyl-L-carnitine.

### 3.12 Identification of acyl-L-carnitine hydrolase from the cell supernatant via biochemical and proteomics techniques

The concentrated cell culture supernatant from the wild type or from the Δ*hyd* strain (see above) were subjected to anion exchange chromatography on a MonoQ 5/50 column. Samples after salt gradient fractionation were assessed for ACHS activity and mass spectrometry was subsequently used for the identification of individual protein constituents. The proteome of fractions with high enzyme activity (~300 mM NaCl eluted) was compared to the proteome of elution samples with negligible ACHS activity (~550 mM NaCl eluted). In addition, a proteomic comparison of the supernatants from cultures grown with acetate versus lauroyl-L-carnitine was carried out. The proteomic approach allowed for the identification of ~200 proteins, respectively. Based on bioinformatics analyses, all cytosolic contaminant proteins as well as proteins without predicted hydrolase activity were excluded (Supplementary Table S2). The overall approach identified a strong candidate: the putative secretory lipase encoded by open reading frame HMPREF0010_02333 featuring a predicted leader peptide of 21 amino acids as indicated by SignalP-6.0 (Teufel et al., 2022) prediction.

### 3.13 Recombinant purification and initial characterization of the acyl-L-carnitine hydrolase candidate protein from the cell supernatant

The following strategy was employed to efficiently overproduce the extracellular *A. baumannii* protein in *E. coli*. The identified protein comprises 435 amino acids and contains two disulfide bonds (Cys104/Cys292 and Cys396/Cys430), as predicted by the AlphaFold 2 protein structure database (entry AF-A0A0R0YLR3-F1-v4). Therefore, the DNA sequence of the target protein was fused to the *pelB* leader sequence from *E. coli* for periplasmic secretion. Furthermore, a C-terminal His-tag sequence was introduced to enable efficient affinity purification. The recombinant overproduction and purification is indicated in Figure 6A. The target protein was purified to apparent homogeneity on Ni^2+^-loaded chelating sepharose using a linear imidazole gradient. Fractions revealed one band with a relative molecular mass of ∼46,000 Da as judged by SDS-PAGE (calculated molecular mass, 46,527 Da). Approximately 15 mg of the purified target protein was obtained per liter cell culture. Analytical size exclusion chromatography revealed a native molecular mass of 47 kDa (Figures 6B,C), indicative for a monomeric quaternary structure.

**Figure 6:**
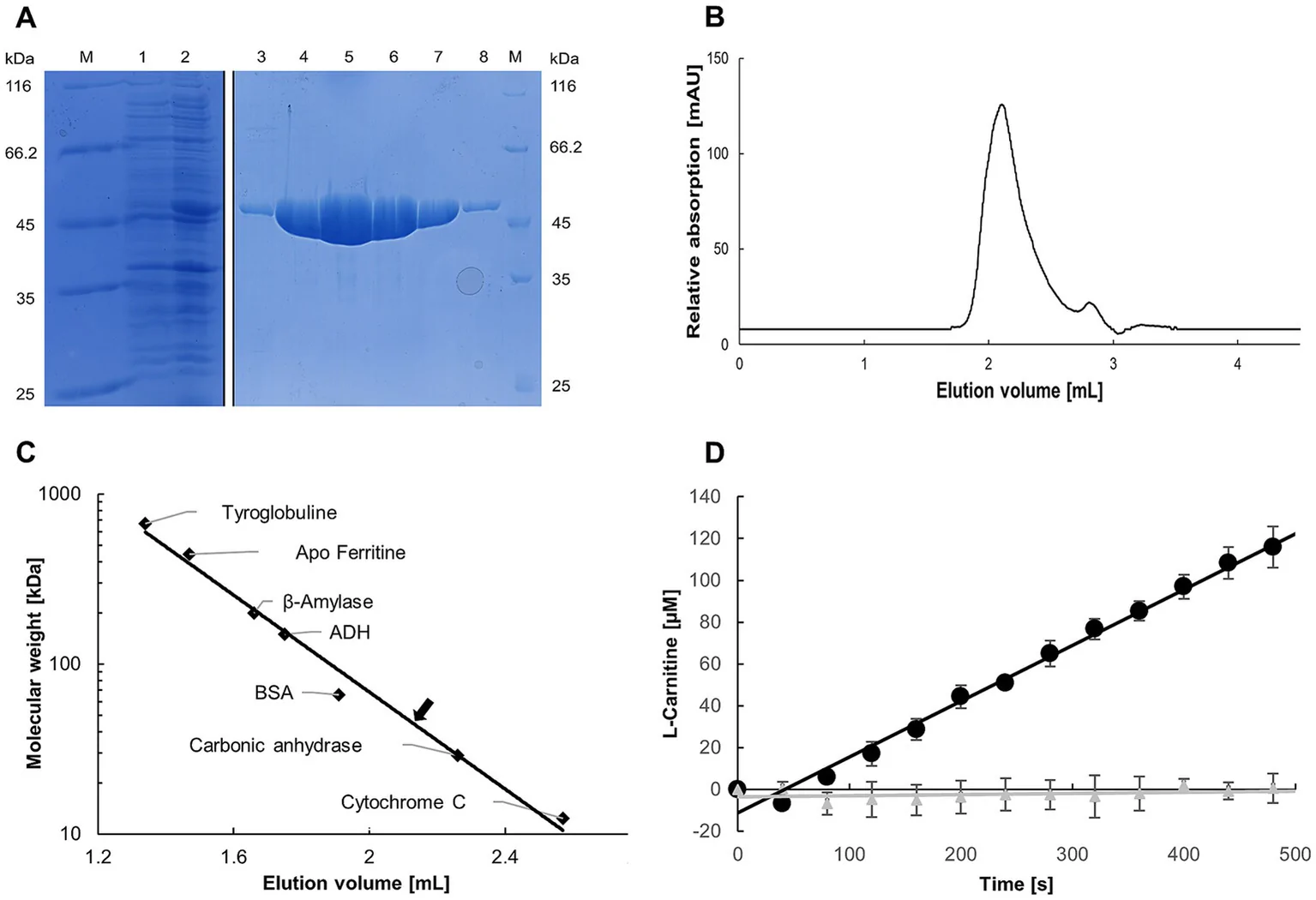
Purification and characterization of the acyl-L-carnitine hydrolase from the supernatant (ACHS). **(A)** SDS-PAGE analysis of the affinity purification of the recombinant acyl-L-carnitine hydrolase. L*ane M*, molecular mass marker, relative molecular masses are indicated; *lane 1 and 2*, *E. coli* cells before and after IPTG induction; *lane 3*–*8*, elution fractions obtained after cell disruption, ultracentrifugation, and affinity chromatography using a linear imidazole gradient on an Äkta explorer chromatography system. **(B,C)** Native molecular mass determination of ACHS by analytical gel filtration. Purified ACHS was analyzed on a Superdex 200 increase 5/150 GL column at a flow rate of 0.45 mL min^−1^ monitoring the absorption at 280 nm. The column was calibrated with thyroglobulin (*Mr* = 660,000), apoferritin (*Mr* = 440,000), β-amylase (*Mr* = 200,000), alcohol dehydrogenase (ADH, *Mr* = 150,000), albumin (BSA, *Mr* = 66,000), carbonic anhydrase (*Mr* = 29,000) and cytochrome c (*Mr* = 12,400). The mass of ACHS is indicated by a black arrowhead. **(D)** Acyl-L-carnitine hydrolase assay containing purified ACHS and palmitoyl-L-carnitine. The liberated L-carnitine was quantified colorimetrically in a continuous spectroscopic assay in the presence of purified L-carnitine dehydrogenase monitoring the formation of NADH at 340 nm (*black curve*). A negative control in the absence of hydrolase was analyzed accordingly (*gray curve*). A slight drift in the negative-control measurements was consistently observed and was attributed to the presence of Triton X100. Each indicated point represents the mean of three independent experiments and error bars represent the standard deviation. All kinetic measurements were reproduced at least two times.

### 3.14 Acyl-L-carnitine hydrolase from the cell supernatant of *A. baumannii* (gene locus tag HMPREF0010_02333) specifically recognizes long-chain acyl-L-carnitines

A coupled lauroyl-L-carnitine hydrolase assay was established for the kinetic investigation of the ACHS candidate protein. L-carnitine dehydrogenase (CdhA, from *P. aeruginosa*) was overproduced and purified (Supplementary Figure S3). Hydrolase activity was monitored in the presence of L-carnitine dehydrogenase and NAD^+^ by spectroscopically quantifying the formation of NADH.

As shown in Figure 6D, the ACHS candidate protein clearly revealed a specific activity of 0.18 ± 0.01 μmol min^−1^ mg^−1^. This experiment unambiguously demonstrates that open reading frame HMPREF0010_02333 encodes the ACHS of *A. baumannii*.

In addition to lauroyl-L-carnitine, the following long-chain substrates were investigated: myristoyl-L-carnitine (C14), palmitoyl-L-carnitine (C16), and stearoyl-L-carnitine (C18). Myristoyl-L-carnitine and stearoyl-L-carnitine revealed clearly increase activities of 3.52 ± 0.13 and 2.09 ± 0.15 μmol min^−1^ mg^−1^. The highest activity of 12.92 ± 1.31 μmol mg^−1^ min^−1^ was determined in the presence of palmitoyl-L-carnitine. All activity assays in the presence of small-chain and medium-chain acyl-L-carnitines (C2-C10) did not reveal detectable hydrolase activity (Figure 7). These experiments clearly indicate that ACHS is a specific hydrolase for the cleavage of long-chain acyl-L-carnitines showing maximum activity in the presence of palmitoyl-L-carnitine. This finding is consistent with the notion that palmitoyl-L-carnitine represents one of the most abundant long-chain acyl-L-carnitines in the human body (Aitken-Buck et al., 2020). As shown in Figures 3, 7, the newly identified ACHS from *A. baumannii* has a complementary substrate spectrum to that of the cytosolic ACH. Our *in vitro* analyses support a metabolic model in which acyl-L-carnitines comprising C12–C18 acyl moieties are hydrolyzed by extracellularly secreted ACHS.

**Figure 7:**
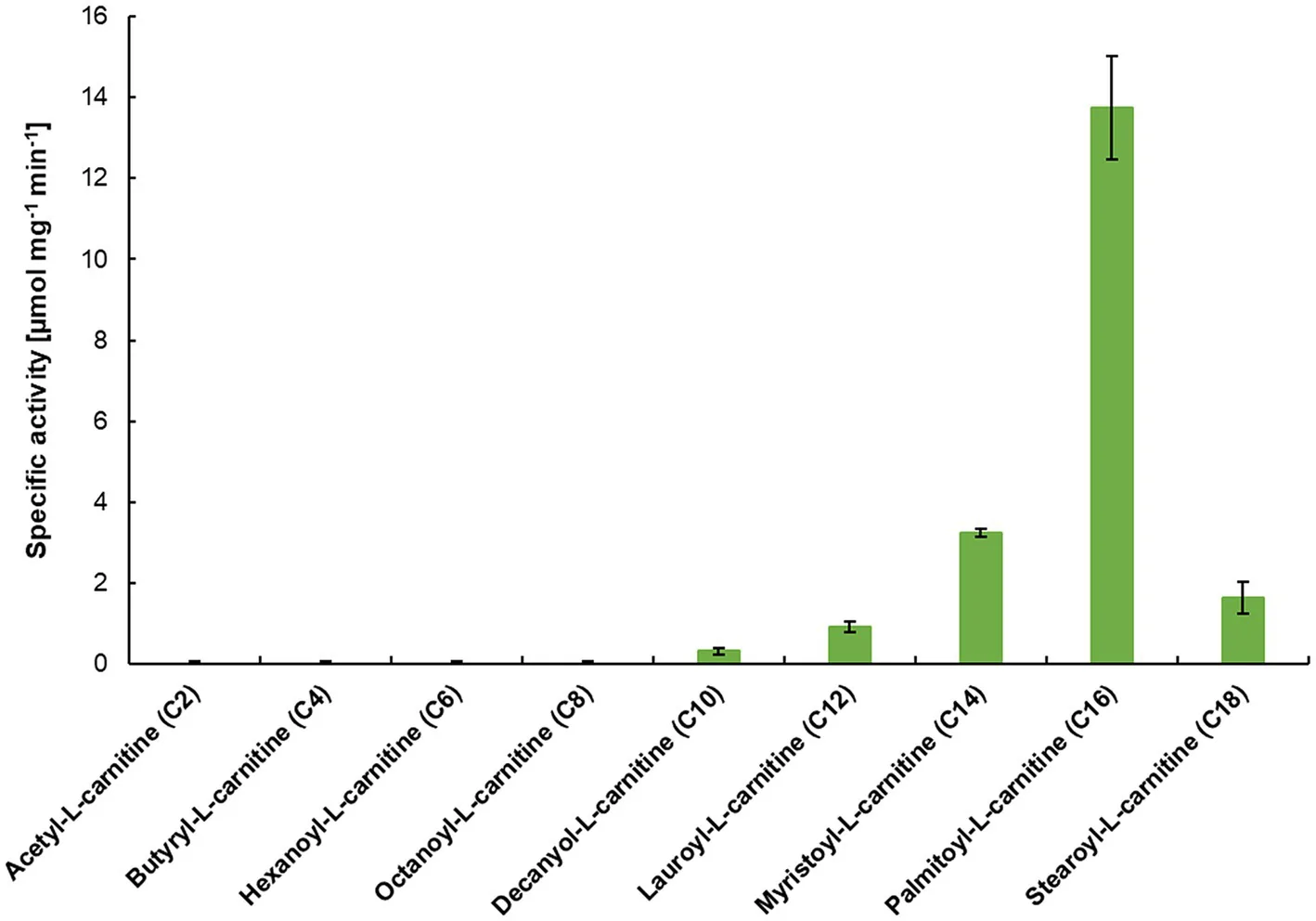
*In vitro* conversion of long-chain acyl-L-carnitines by ACHS. The specific activity of purified ACHS in the presence of the indicated substrates was calculated (*green bars*). Each data point corresponds to the mean of three independent experiments, with error bars representing the standard deviation. ACHS did not reveal detectable enzymatic activity in the presence of acetyl-L-carnitine, butyryl-L-carnitine, hexanoyl-L-carnitine and octanoyl-L-carnitine. Error bars indicate the detection limit of the employed assay (0.05 μmol mg^−1^ min^−1^).

## 4 Discussion

This study sheds light on the versatile metabolism of *A. baumannii* when utilizing different acyl-L-carnitines. These molecules exhibit a chain length-dependent propensity to form micelles, with small-chain and medium-chain acyl-L-carnitines being more soluble. In contrast, the long-chain molecule palmitoyl-L-carnitine has a critical micelle concentration around 10 μM (Liu et al., 2022). These biophysical properties, particularly the differences in molecular size among these molecules, may necessitate distinct biochemical strategies for their metabolism.

Infection assays in *Galleria mellonella* larvae using the Δ*hyd* mutant revealed that the acyl-L-carnitine metabolism contributes to the virulence of *A. baumannii* (Breisch et al., 2022). At the same time, acyl-L-carnitines are key molecules that facilitate fatty acid degradation in human mitochondria. These observations therefore suggest that the energy metabolism examined in this study may also be relevant during human *A. baumannii* infections.

Within the L-carnitine degradation operon, the hydrolase encoded by HMPREF0010_01348 was identified as a cytoplasmic ACH that hydrolyzes small-chain and medium-chain acyl-L-carnitine molecules. Growth assays with the Δ*bcct* mutant on small-chain and medium-chain acyl-L-carnitines confirmed that the BCCT transporter mediates uptake of these substrates.

In *Pseudomonas aeruginosa*, utilization of acyl-L-carnitines containing C2–C16 acyl groups, with the exception of octanoyl-L-carnitine, has been reported in the literature. A cytoplasmic acyl-L-carnitine hydrolase encoded by open reading frame PA5384 (*hocS*) was identified as essential for growth on and hydrolysis of acetyl- and butyryl-L-carnitine (Meadows and Wargo, 2013). Evidently, this enzyme exhibits a distinct substrate specificity favoring shorter acyl chains compared to the ACH from *A. baumannii*. This difference was not predictable from sequence analysis, as the two hydrolases share a sequence identity of 42%. Based on *P. aeruginosa* growth data, the presence of a second acyl-L-carnitine hydrolase with preference for long-chain substrates has been proposed; however, a genetic screening and transcriptome-guided search did not facilitate the identification of such enzyme.

During growth of *A. baumannii* on acetyl-L-carnitine, transient extracellular accumulation of L-carnitine indicated initial utilization of acetate, followed by L-carnitine catabolism in later growth phases. Although purified ACH showed no activity toward long-chain substrates, both wild type and Δ*hyd* strains grew on these compounds, suggesting the presence of an additional hydrolase. Enzymatic activity assays using the culture supernatant led to identification of open reading frame HMPREF0010_02333 which encodes for a second acyl-L-carnitine hydrolase that is secreted in the cell supernatant. This ACHS efficiently cleaved long-chain acyl-L-carnitines.

The findings of the present study provide the basis for the metabolic model depicted in Figure 8. The transporter BCCT functions as a discriminator among the various acyl-L-carnitine substrates. Small-chain and medium-chain substrates are transported into the cell via this transporter and are subsequently cleaved by cytosolic ACH which is encoded in the L-carnitine degradation gene cluster. The resulting alkanoic acids can be converted into acetyl-CoA which enters the TCA cycle. This β-oxidation cycle, produces NADH and FADH_2_ and involves the enzymes acyl-CoA synthetase FadD, acyl-CoA dehydrogenase FadE, 2-enoyl-CoA hydratase FadB, L-3-hydroxyacyl-CoA dehydrogenase FadB and 3-ketoacyl-CoA thiolase FadA (Pavoncello et al., 2022).

**Figure 8:**
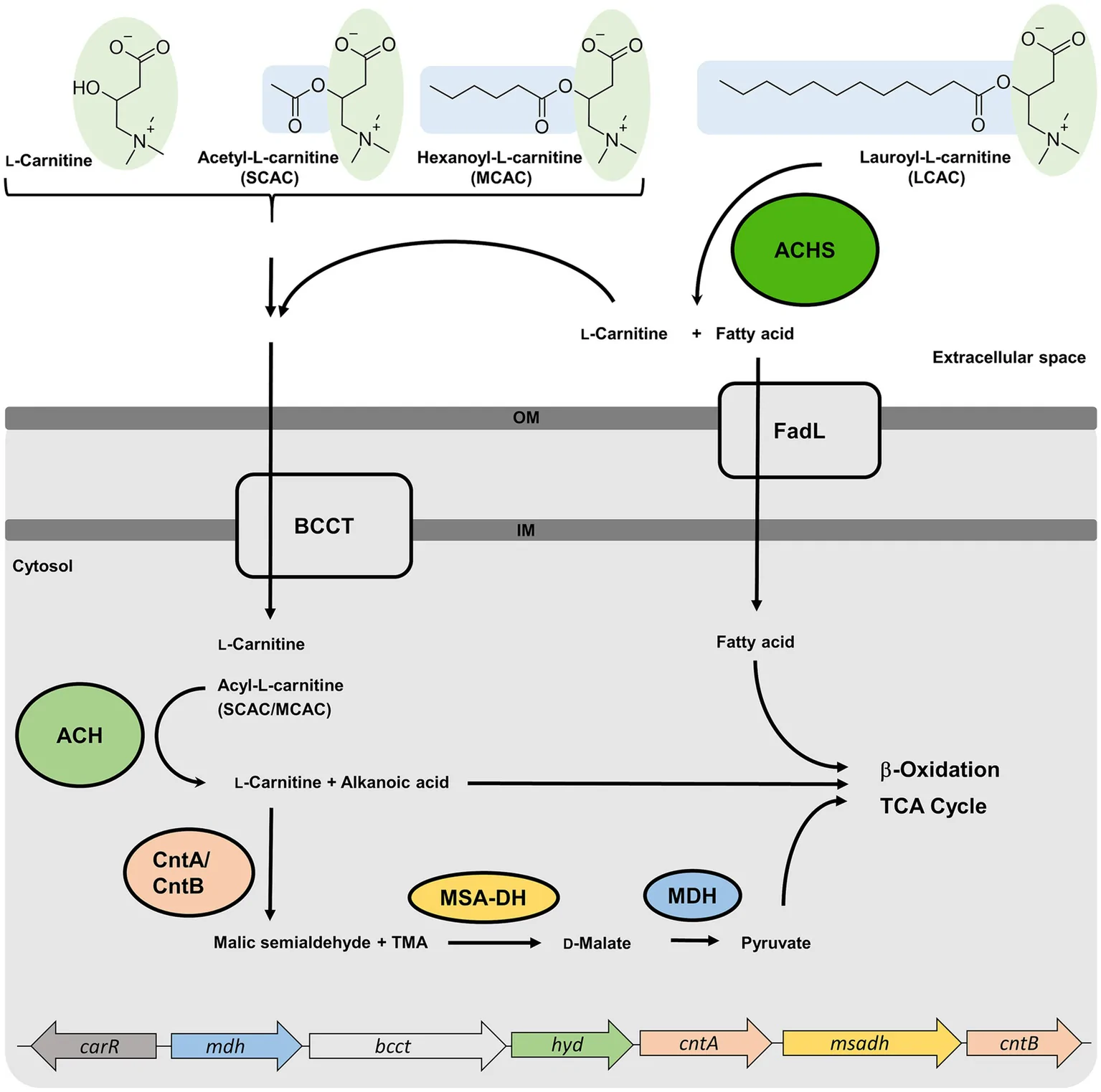
Overview of the acyl-L-carnitine metabolism in *A. baumannii*. Short-chain, medium-chain, and long-chain acyl-L-carnitines (SCAC, MCAC, and LCAC) serve as carbon and energy sources for *A. baumannii*. L-carnitine, acetyl-L-carnitine, and hexanoyl-L-carnitine are translocated into the cytosol via a betaine/choline/carnitine transporter (BCCT). Intracellular acyl-L-carnitine hydrolase (ACH) then facilitates for the cleavage into L-carnitine and an alkanoic acid. The latter molecule is then channeled (via β-oxidation) into the TCA cycle. L-carnitine is first converted into malic semialdehyde by carnitine monooxygenase (CntA/CntB) which is then transformed into D-malate by malic semialdehyde dehydrogenase (MSA-DH). Finally, the central metabolite pyruvate is formed by the action of malate dehydrogenase (MDH). Lauroyl-L-carnitine is not a substrate of BCCT. An extracellular acyl-L-carnitine hydrolase (ACHS) enables for the hydrolysis of lauroyl-L-carnitine in the cell supernatant. The resulting fatty acid is translocated into the cytosol via the FadL transporter, while L-carnitine enters the described degradation pathway.

The remaining genes within the *A. baumannii* carnitine degradation cluster encode the enzymes carnitine monooxygenase (CntA/CntB), malic semialdehyde dehydrogenase (MSA-DH) and D-malate dehydrogenase (MDH) (Breisch et al., 2019; Massmig et al., 2020; Piskol et al., 2022). Carnitine monooxygenase facilitates the conversion of L-carnitine into trimethylamine and malic semialdehyde. The latter molecule is subsequently transformed into the D-enantiomer of malate, whose stereochemistry impedes direct entry into the tricarboxylic acid cycle. Therefore, the further conversion of D-malate into pyruvate is required. Pyruvate is channeled into the tricarboxylic acid cycle, where it contributes to energy metabolism and anabolic pathways.

Due to their biophysical and spatial constraints, long-chain acyl-L-carnitines cannot be transported into the cell via the BCCT transporter. Extracellular hydrolysis of these substrates is required to facilitate their subsequent cellular uptake. The newly identified ACHS possesses a leader sequence that most likely enables the secretion of the protein via the Sec translocation machinery. The genomic context of the ACHS gene encodes no proteins related to L-carnitine or fatty acid metabolism. The extracellular ACHS enzyme exhibits remarkable stability, as demonstrated by the fact that incubating the recombinant protein at 99 °C for 15 min does not result in complete inactivation. Such robustness is commonly observed in extracellular enzymes, including lipases (Ali et al., 2023). ACHS possesses maximal enzymatic activity in the presence of palmitoyl-L-carnitine, while it lacks any detectable activity toward short- and medium chain substrates (Figure 7). Our growth experiments on long-chain acyl-L-carnitines showed an immediate accumulation of L-carnitine in the culture supernatant (Figure 5B), with a continuous decrease of L-carnitine observed only during the late exponential phase. These measurements indicate that, also in the case of long-chain acyl-L-carnitines, the fatty acid is metabolized initially, while the catabolism of carnitine occurs during a later growth phase. Thus, for short-, medium-, and also for long-chain acyl-L-carnitines, the respective carboxylic acids are the preferred substrates for the metabolism of *A. baumannii*. Such diauxic growth was also suggested from the curve progression in our plate reader cultivation experiments (compare Figure 4A).

In bacteria, long-chain fatty acids are taken up via specialized transporters, with FadL being the primary outer membrane protein responsible. FadL is a 48 kDa β-barrel protein that recognizes long-chain fatty acids at its extracellular surface and facilitates their translocation into the periplasm. The mechanism of transport across the periplasm and inner membrane is less clear. At the inner membrane, long-chain fatty acids are believed to cross the phospholipid bilayer via a spontaneous flip-flop mechanism which is followed by their activation through thioesterification by the cytosolic acyl-CoA synthetase FadD (Kamp et al., 1995; Pavoncello et al., 2022). The second product of the ACHS-catalyzed reaction is L-carnitine, which is transported into the cell via the BCCT transporter. Once inside, L-carnitine undergoes metabolism through the degradation pathway outlined above.

Recently, bacterial trimethylamine (TMA) production has gained significant interest owing to its clinical importance. This gut microbiota-derived metabolite has been linked to heightened cardiovascular disease risk. Dietary nutrients like carnitine and choline are converted to TMA by gut bacteria, absorbed through the intestinal epithelium, and then oxidized to trimethylamine oxide (TMAO) by liver flavin monooxygenases. Multiple studies report a positive correlation between elevated plasma TMAO concentrations and overall mortality, especially from cardiovascular and renal disease (Kanitsoraphan et al., 2018; Chen et al., 2022). The gut microbiota derived production of TMAO has been evaluated through the presence of key TMA-generating enzymes, including choline-TMA lyase (CutC), betaine reductase (GrdH), and carnitine monooxygenase (CntA) (Falony et al., 2015; Rath et al., 2017; Rath et al., 2019). Based on the present investigation, the concurrent presence of, e.g., CntA/CntB together with ACH- and ACHS-encoding genes may enable the synthesis of considerable amounts of TMA. However, further studies are needed to quantitatively determine the relative contributions of ACH and ACHS to TMA production within a specific organism.

## Data Availability

The original contributions presented in the study are included in the article/Supplementary material, further inquiries can be directed to the corresponding author.
