# Supplementary material for: Metabolic versatility of Acinetobacter baumannii: distinct pathways for the utilization of short-, medium- and long-chain acyl-L-carnitines
Source: Front Microbiol. 2026 Sep 18;17:1964901. doi: 10.3389/fmicb.2026.1964901 (PMC13631058; doi:10.3389/fmicb.2026.1964901)
Supplement: Supplementary file 1 [file Supplementary_file_1.DOCX]

***Supplementary Material***

**1	Supplementary Figures and Tables**

**Figure S1.** **Growth of *A. baumannii* strains on various acyl-L-carnitines*.*** The growth of the *A. baumannii* wild type, Δ*bcct* and Δ*hyd* strain was assessed on MOPS minimal medium supplemented with acyl-L-carnitines (5 mM) or acetate (5 mM) as carbon and energy source. The optical density at 600 nm (OD_600_) was measure using a microplate reader. At least three independent experiments were conducted and one representative growth curve is depicted.

**Figure S2.** **ACH assays using substrates with different acyl chain lengths*.*** Enzyme assays were performed in the presence of 200 nM of recombinant purified hydrolase using 300 µM of the respective substrates: buturyl-L-carnitine, hexanoyl-L-carnitine, octanoyl-L-carnitine, lauroyl-L-carnitine and palmitoyl-L-carnitine (*black lines*). L-carnitine concentrations were determined spectrophotometrically as detailed in the methods section. Control experiments with heat-inactivated enzymes were performed accordingly (*grey lines*). The means of three independent experiments were plotted against time, and the standard deviation is given.

**Figure S3.** **Purification of L-carnitine dehydrogenase.** SDS-PAGE analysis of the affinity purification of L-carnitine dehydrogenase from *P. aeruginosa*. *Lane* *M*, molecular mass marker, relative molecular masses are indicated. *Lane 1 and 2*, *E. coli* cells before and after induction; *lane 3*, cell-free extract after cell disruption and ultracentrifugation; *lanes 4 and 5*, column flow through; *lanes 6 - 9*, elution fractions after affinity chromatography.

**Figure S4.** **ACHS assays using substrates with different acyl chain lengths*.*** Enzyme assays were performed in the presence of 25 – 160 nM of recombinant purified hydrolase using an acyl-L-carnitine concentration of 160 nM, respectively. A continuous spectroscopic assay was performed in the presence of purified L-carnitine dehydrogenase and 2 mM NAD^+^ by monitoring the formation of NADH at 340 nm (*black line*). Negative controls in the absence of hydrolase were analyzed accordingly (*grey line*). The means of three independent experiments were plotted against time, and the standard deviation for selected time points is given.

**Table S1: Synthetic genes and oligonucleotides**

| **Sequence** | **Name** | **Construct** |
| --- | --- | --- |
| GTTGGATGGTTGGTGGCCTGGATAGCCATGAGTTTATTACCAGCTATCTGTGCAAAGATCTGAATGCCGTTGTTATCAGCGTTGATTATCGTCTGGCACCGGAACATCGTTTTCCGGCAGCATTTGAAGATTGTCTGGCCGTTTATCATTGGCTGAAACAGCATGGTAGCGCATGGCAGATTGATAGTGAAAATATTGTTCTGGCAGGCGATAGTGCCGGTGGTAATCTGGCAGCAGCACTGGCAGTTGAACTGCAGCATAGCGGTCTGCAGGCACAAGGTCTGGCACTGGTTTATCCGTGTCTGACCACCGCATTTGATACCGCAAGCGCACAGAAACATGCACATGCACCGCTGCTGACCACAGAAGATATGCATTTTTATCTGAAAGAGTATGCACCGGATAGCCAGGATTGGCAGGATCTGCGCCTGGCTCCGCTGCTGGCAACCGATTTTAGCGATATGCCGACCAGCTTTGTTGCAGTTGCAGAATATGATCCGCTGAGTGATGATGGTTATTTTCTGACCCAGAAACTGGAACAGGCAGGTATTCCGAATGAATTTCATTTAGGTAAAGGTCTGCTGCATGGTAGTCTGCGTCTGATGCGTGATTGTCCGGAAGTTCAGCATCTGTATCAGAATATGCTGAGCGCAATTCGTCGTATGCTGAGTAGCGCAGAGAAAACCAGCATTTAAGCGGCCGCAC | *ach* | pETM11_*ach* |
| GATCCATATGAAATACCTGCTGCCGACCGCTGCTGCTGGTCTGCTGCTTCTCGCTGCCCAGCCGGCGATGGCCGCCATGGGCAACGATGATGATGACGATTATATTGGTGTGAACCCGGATAAAACCTACATCAGCGAAAAGAATTATAGCCAGGATACCCTGAGCGGTGCAGCAAGCATTAAAGTTATGACCTATAACATGGAAAACGTGCAGGGTAAAACCGCACAGGCAACCGCACTGGTTATGTTTCCGAAAGTTACCCAGCCGAAAGATGGTTATCGTGTTGTTGTTTGGGAACATGGCACCGTTGGTGTTGGTGATAGCTGTGCACCGAGCAATAATACCATTAATCCGCGTTTCAAAATTCTGGCAGATACCCTGCTGGCAGCCGGTTATGTTGTTATTGCACCGGATTATGAAGGTCTGGGCACCCGTGGTATGCATCCGTATCTGAATCTGAGCAGCGAAGCAAAAAGCGCACTGGCAGCAGTTAAAGCAGCAAAAGATCATTATGGCAATCAGCTGAATGGTGCATGGATGAGCATTGGTCAGAGCCAAGGTGGTCATGCAAGCCTGGGCACCGCAGAATTTGCGAATAATGATGCAAACTATAAAGGTGCAGTTGCGGGTGCACCGGCATCTAGCCTGGGTTATATCATTAGCACCGTTGCACCGCAGGCAATTCAGGATATTCTGAAGAAAGAACAGGCAGGCACCGTTCCGGTTGGTACAGCAGTTGAAGTTTATGCAGAACTGCTGGCCTATGCAGCATATACCACCGTGGGTATTACCGCCTATGAACCGAAATTTAACTATCGCGAAATTTTTCAGCAGCGCAGCCAGAGCATTGCCGAATTTGCCGAAGGCACCACCGGTGAAAATGGTGTTTGTCTGACCGATCTGCATAACAAATTTGCGGATGATATTCGTGATTTTCTGGCAACCAATGCAGGCAAAACCGTTCTGGATTATCCTGGTCTGGCAGGTAATTTTCAAGAAAATCCGACCGTGAAAAAGTTCCTGGTTGATAATCAGCCTGCCACCAAAAAGATTAATAGTCCGGTGATGATTGTTCAGGGTACAGCCGATATGGCAGTTCCGTATCCGGTTACCGATGCACTGCAGAATGGTCTGAAAAAGATGGGCACCGATGTTACCTTTGTTCCGGTTCTGGGTGCAGCACATACCCAGGCAATTGTTTGTCGTAATGCAGAAATCTATCAGTTCGTGCAGAGCAAAATGCCTGCCAAAACCAATATTGTTCTGGATCCGAGCGTTATTGATGCCAGCAAAAATGTTGAATGTACCGGTATTGCACAAGCGGCCGCGATC | *achs* | pETM50_pelB_*achs* |
| GATCCCATGGGAATGTCCTTCGTCACCGAGA | Primer 1 | pBAD_His_AM_cdhA |
| GATCGCGGCCGCTCACTCGGCGAAGGCGAAG | Primer 2 | pBAD_His_AM_cdhA |

**Table S2: Identification of potential acyl-L-carnitine hydrolase proteins from the culture supernatant of *A. baumannii***

The cell culture supernatant from the *A. baumannii* Δ*hyd* strain was concentrated and fractionated using anion exchange chromatography on a MonoQ 5/50 column. Mass spectrometry was used to compare samples showing high ACHS activity (C2 and C3, eluted at ~300 mM NaCl) with a sample devoid of ACHS activity (C6, eluted at ~550 mM NaCl). The number of identified peptides, sequence coverage, relative abundance, and molecular mass of the respective proteins are indicated. Only the candidates with a postulated hydrolase function are shown among the ~200 proteins identified.

| **Locus or short name** | **Sequence coverage of peptides**  **[%]** | **Identified**  **Peptides** | **Relative abundance**  **Fraction C2** | **Relative abundance**  **Fraction**  **C3** | **Relative abundance**  **Fraction**  **C6** | **Molecular**  **Mass [Da]** | **Proposed function** |
| --- | --- | --- | --- | --- | --- | --- | --- |
| HMPREF0010_01942 | 3.08 | 1 | 2.41E+04 |  |  | 33756 | Hydrolase, alpha/beta domain protein |
| ggt | 8.17 | 6 | 2.94E+05 |  |  | 69792 | Glutathione hydrolase proenzyme |
| fghA | 3.44 | 1 | 5.20E+04 |  |  | 32647 | S-formylglutathione hydrolase |
| HMPREF0010_02333 | 20.23 | 8 | 2.11E+06 | 1.58E+06 | 5.14E+05 | 46391 | Secretory lipase |
| HMPREF0010_01064 | 5.63 | 1 | 3.44E+05 | 5.40E+04 |  | 24970 | Uncharacterized protein |
| HMPREF0010_02862 | 17.05 | 6 | 3.94E+05 | 2.41E+05 | 2.43E+04 | 32438 | Phospholipase, patatin family |
